# Propofol-Induced Hyperglycemia in the Critically Ill: An Unfamiliar Side Effect of a Common Anesthetic

**DOI:** 10.7759/cureus.74263

**Published:** 2024-11-22

**Authors:** Muhammad A Qureshi, Greeshma A Thomas, Tijin Mathew, FNU Anshul

**Affiliations:** 1 Internal Medicine, Southeast Health Medical Center, Dothan, USA; 2 Critical Care Medicine, Southeast Health Medical Center, Dothan, USA

**Keywords:** hyperglycemia, intensive care unit, mechanical ventilation, propofol, sedation

## Abstract

Hyperglycemia is associated with increased in-hospital morbidity and mortality, especially in critically ill intensive care unit (ICU) patients. Propofol, a common anesthetic used in the ICU, may cause hyperglycemia by inducing insulin resistance, reducing insulin-stimulated glucose uptake in muscles, and attenuating insulin-mediated suppression of hepatic glucose. We present the case of a 58-year-old female who was admitted for sepsis secondary to cellulitis but required intubation for respiratory failure. She was provided propofol for sedation and went on to develop propofol-induced hyperglycemia. This is one of the few documented human cases demonstrating the association between propofol and hyperglycemia. There are animal-based studies that demonstrate this effect as well. This case report highlights the fact that propofol-induced hyperglycemia should be a consideration when deciding sedation strategies in critically ill patients.

## Introduction

According to Yasuda et al., hyperglycemia in critically ill patients is linked to increased susceptibility to infection, renal insufficiency, prolonged ventilator dependence, extended intensive care unit and hospital stay, as well as higher mortality rates [1]. Propofol is a commonly used intravenous anesthetic in the critical care setting and may lead to hyperglycemia. According to Sahinovic et al., propofol enhances the inhibitory effects of gamma-aminobutyric acid in the central nervous system [2]. Although human studies on propofol’s impact on glycemic control are limited, research such as the euglycemic hyperinsulinemic clamp study in rats under propofol anesthesia and conscious unrestrained rats suggests that propofol may induce hyperglycemia. Propofol is thought to induce insulin resistance, reduce insulin-stimulated glucose uptake in skeletal and cardiac muscle, and attenuate insulin-mediated suppression of hepatic glucose output in rats [1]. Here, we report a rare case of propofol-induced hyperglycemia in a critically ill patient who was admitted for sepsis with multiple sources and respiratory distress.

## Case presentation

A 58-year-old female with a past medical history of hypertension, type 2 diabetes mellitus on insulin glargine 15 units nightly with medication non-compliance, non-alcoholic cirrhosis associated with thrombocytopenia and coagulopathy, rheumatoid arthritis, and lung cancer status post-radiotherapy and immunotherapy in 2022 was admitted for urosepsis, respiratory distress, and bilateral knee amputation stump cellulitis. Given her prior history of extended-spectrum beta-lactamase (ESBL) urinary tract infection (UTI), she was empirically started on meropenem and vancomycin. She was also noted to be in respiratory distress on arrival and was placed on Optiflow. On admission, her random blood glucose was 511 mg/dL, which was due to non-compliance with her insulin and empagliflozin. Her last two hemoglobin A1c readings were 10.9% and 12.1%. She was started on a glucose stabilizer which resulted in an improvement of her glucose and was subsequently transitioned to insulin glargine and insulin aspart.

Her urine and blood culture revealed the growth of ESBL *Escherichia coli* and she started to develop worsening respiratory distress for which she was transitioned to non-invasive ventilation. The development of acute encephalopathy further complicated her hospital course. A chest X-ray was obtained and demonstrated left upper lobe atelectasis. Given altered mentation and respiratory failure, the patient was intubated and placed on invasive mechanical ventilation on day seven of admission.

**Figure 1:**
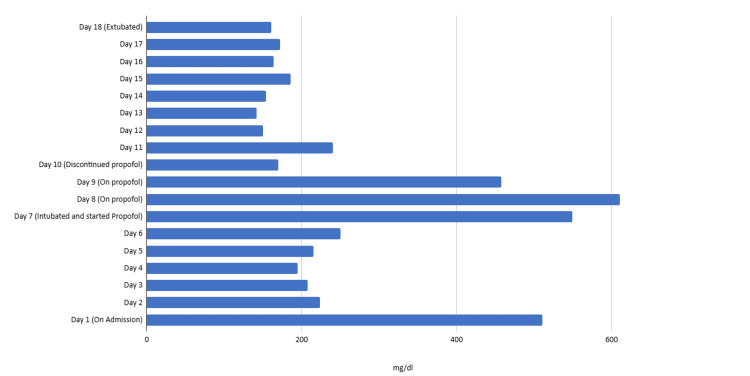
Patient’s blood glucose level trends in mg/dL.

From day two to three, her blood glucose ranged from 140 to 250 mg/dL and she was started on insulin glargine 30 units twice daily along with sliding scale insulin with a target glucose of 140-180 mg/dL. After intubation, she was started on sedation with propofol, and it was observed that blood glucose started spiking thereafter. On day eight, her blood glucose was 611 mg/dL and her insulin glargine was increased from 30 to 50 units twice daily along with the sliding scale insulin. On day nine, her blood glucose remained persistently elevated at 458 mg/dL, raising concerns for propofol-induced hyperglycemia. Given the concern for propofol-induced hyperglycemia, propofol was discontinued and she was started on Precedex for sedation. On day 10, after the discontinuation of propofol, her blood glucose improved to 170 mg/dL. The patient’s glucose trends are demonstrated in Figure 1. Given this improvement, her insulin glargine was decreased to 40 units twice daily and then to 30 units twice daily. The patient’s encephalopathy and respiratory status improved significantly and she was successfully weaned off of all sedation. The patient was extubated on day 18 of hospitalization. She was discharged home with insulin glargine 15 units nightly and educated about the importance of compliance with a diabetic diet and medications.

## Discussion

Hyperglycemia due to propofol is not well documented in the literature involving humans. A retrospective analysis by Kim et al. is one of the few studies demonstrating this effect. This study compared sevoflurane and propofol anesthesia to the incidence of hyperglycemia in patients with type 2 diabetes undergoing lung surgery [3]. The study demonstrated persistent hyperglycemia in patients who were provided anesthesia with propofol [3]. This is a notable side effect given that the ideal blood glucose level in the critically ill population is 140-180 mg/dL [4].

Multiple animal-based studies have demonstrated the relationship between propofol and hyperglycemia. One study demonstrated a decrease in insulin sensitivity by about 50% in canine subjects who were provided initial anesthesia with propofol and maintenance anesthesia with inhaled isofluorane or sevoflurane [5]. Another study by Yasuda et al. demonstrated similar results [1]. In their study, anesthesia with propofol caused insulin resistance and a reduced effect of insulin-mediated suppression of hepatic glucose output in rats [1]. Li et al. also reported that propofol, due to its hydrophobic nature and high lipid content, can exaggerate insulin resistance [6]. Propofol was found to be the major cause of hyperinsulinemia and insulin resistance in the study [6].

This relationship between propofol and hyperglycemia is important to be aware of as it can change our sedation strategies in the critically ill, especially in diabetic patients. Propofol has been a cornerstone for sedation in critically ill patients until recently with the introduction of fentanyl-based analgesosedation [7]. With this discovery, more physicians may consider switching from a propofol-based strategy to a fentanyl-based strategy. Data supporting these findings are limited to a few studies, and larger clinical trials should be performed to confirm our findings.

## Conclusions

Propofol-induced hyperglycemia should be a consideration when deciding sedation strategies in critically ill patients. Although not well documented in the literature, this effect can negatively impact patient outcomes. Our case demonstrated a correlation between propofol and hyperglycemia and is supported by one human trial and a few animal trials. One important factor to consider is that our patient was diabetic, and this relationship has not been studied in patients without diabetes. Underlying sepsis may have also been a contributing factor in our patient’s hyperglycemia. However, there was a clear correlation between the initiation of propofol and the onset of hyperglycemia. More research is required to confirm our findings as current literature is limited.
